# Postharvest chilling diminishes melon flavor *via* effects on volatile acetate ester biosynthesis

**DOI:** 10.3389/fpls.2022.1067680

**Published:** 2023-01-06

**Authors:** Huijun Zhang, Xiuxiu Zhu, Runzhe Xu, Yushu Yuan, Modesta N. Abugu, Congsheng Yan, Denise Tieman, Xiang Li

**Affiliations:** ^1^ School of Life Science, Huaibei Normal University, Huaibei, Anhui, China; ^2^ Horticultural Sciences, North Carolina State University, Raleigh, NC, United States; ^3^ Horticultural Institute, Anhui Academy of Agricultural Sciences, Hefei, China; ^4^ Horticultural Sciences, Genetics Institute, University of Florida, Gainesville, FL, United States

**Keywords:** melon, chilling, fruit flavor, volatile acetate esters, transcriptome, alcohol acyltransferase

## Abstract

In postharvest handling systems, refrigeration can extend fruit shelf life and delay decay *via* slowing ripening progress; however, it selectively alters the biosynthesis of flavor-associated volatile organic compounds (VOCs), which results in reduced flavor quality. Volatile esters are major contributors to melon fruit flavor. The more esters, the more consumers enjoy the melon fruit. However, the effects of chilling on melon flavor and volatiles associated with consumer liking are yet to be fully understood. In the present study, consumer sensory evaluation showed that chilling changed the perception of melon fruit. Total ester content was lower after chilling, particularly volatile acetate esters (VAEs). Transcriptomic analysis revealed that transcript abundance of multiple flavor-associated genes in fatty acid and amino acid pathways was reduced after chilling. Additionally, expression levels of the transcription factors (TFs), such as *NOR*, *MYB*, and *AP2*/*ERF*, also were substantially downregulated, which likely altered the transcript levels of ester-associated pathway genes during cold storage. VAE content and expression of some key genes recover after transfer to room temperature. Therefore, chilling-induced changes of VAE profiles were consistent with expression patterns of some pathway genes that encode specific fatty acid- and amino acid-mobilizing enzymes as well as TFs involved in fruit ripening, metabolic regulation, and hormone signaling.

## Introduction

Melon is one of the most important and popular horticultural crops worldwide because of its excellent flavor and rich nutrition (Pereira et al., 2018; Yano et al., 2020; Zhang et al., 2021). The unique flavor of melon fruit (*Cucumis melo* L.) is a sum of interactions among soluble sugars, acids, and various volatile organic compounds (VOCs). VOCs are critical contributors to fruit quality and consumer preference (Schwab et al., 2008). In climacteric melon, approximately 240 VOCs have been identified, including aldehydes, alcohols, terpenes, esters, and sulfur-containing aroma compounds (Beaulieu and Grimm, 2001; Fallik et al., 2001; El-Sharkawy et al., 2005; Kourkoutas et al., 2006; Obando-Ulloa et al., 2008; Gonda et al., 2016; Esteras et al., 2018). High levels of sulfur-containing compounds may cause undesirable aromas in melon fruit (Gonda et al., 2013). Volatile esters, generally described as fruity or floral-like, are the most abundant VOCs and make a positive contribution to melon flavor and consumer liking (Gonda et al., 2016; Esteras et al., 2018). In contrast, non-climacteric melons show a lack of esters (Aubert and Pitrat, 2006; Sangster et al., 2006; Obando−Ulloa et al., 2008).

The flavor-related VOCs are mostly derived from amino acid, fatty acid, and carotenoid precursors (Schwab et al., 2008; Klee and Tieman, 2018). Most of the VOCs in climacteric melon are esters derived from fatty acids and amino acids (Gonda et al., 2016). Multiple flavor-impacting genes have been identified in melon. In the fatty acid pathway, lipoxygenases (LOXs) are classified into two groups, including 9-LOX and 13-LOX, generating 9(*S*)- and 13(*S*)-hydroperoxides of linoleic and linolenic acid. Hydroperoxide lyases (HPLs) can catalyze 13(*S*)-hydroperoxides to generate corresponding C6 aldehydes (2017; Chen et al., 2004; Shen et al., 2014; Zhang et al., 2015; Zhang et al., 2017). Additionally, pyruvate decarboxylases (PDCs) are prominent enzymes and associate with the decarboxylation of keto-acids generating acetaldehyde, propanal, pentanal from their corresponding carboxylic acids (Wang et al., 2019). In the amino acid pathway, branched-chain amino transferases (BCATs) and aromatic amino transferases (ArATs) are involved in the initial step in the formation of amino acid-derived aldehydes (Gonda et al., 2010). Alcohol dehydrogenases (ADHs) can catalyze the reduction of aldehydes to generate alcohols (Speirs et al., 1998; Chen et al., 2016). Esters are the most important VOCs that contribute to melon flavor, and they are mainly derived from fatty acids and amino acids (Gonda et al., 2016). The most abundant esters include acetate, butyrate, hexanoate and benzoate esters of fatty acids and amino acids, with acyl-coenzyme A (2 carbons), butyryl-CoA (4 carbons), hexanoyl-CoA (6 carbons) and benzoyl-CoA (7 carbons) as the co-substrate, respectively. In the final step, alcohol acyltransferases (AATs) can catalyze the esterification of an acyl moiety from an acyl-CoA donor onto alcohol, to form the corresponding esters (Goulet et al., 2015; Peng et al., 2020). In addition to formation of esters, degradation is also an important step to balance ester levels in fruit. In tomato, carboxylesterases (CXEs) catalyze the removal of the ester group to release the corresponding alcohol (Goulet et al., 2012; Cao et al., 2019).

In addition to the biosynthetic enzymes directly involved in production of volatile esters, transcription factors (TFs) also play key roles in the biosynthesis of flavor-associated VOCs in fruit crops. *Colorless nonripening* (*CNR*) and *nonripening* (*NOR*) genes are important fruit development and ripening regulators in plants. In tomato, levels of the fatty acid-derived VOCs in the ripening *cnr* and *nor* mutants are substantially lower than those in the wild-type (Zhang et al., 2016; Gao et al., 2022). Hormones also play important roles in fruit VOC biosynthesis *via* impacting the expressions of VOC-associated genes (Jin et al., 2016). An ethylene-responsive AP2/ERF family member, CitERF71 was associated with volatile terpene synthesis by activating transcription of a terpene synthase gene (*CitTPS16*) in sweet orange (Li et al., 2017). An ERF#9-MYB98 complex is involved in furaneol production *via* transactivation of quinone oxidoreductase *FaQR* (Zhang et al., 2018). For flavor-associated esters, MYB and bZIP have been shown to interact with the promoters of ester-related AATs in apple and banana, respectively (Li et al., 2014; Guo et al., 2018). Additionally, recent research revealed a peach NAC transcription factor homologous to tomato NOR (*PpNAC1*), positively regulates ester biosynthesis *via* activation of *PpAAT1* (Cao et al., 2019). In melon, a ripening regulator, CmNOR involved in ethylene production also activates the production of esters (Santo Domingo et al., 2022).

Deterioration in fruit flavor quality has become a major cause of consumer dissatisfaction (Whiteside. 1977; Bruhn et al., 1991; Fernqvist and Hunter., 2012; Klee and Tieman, 2018; Colantonio et al., 2022). For example, modern commercial tomato varieties are generally substantially less flavorful than heirloom varieties. Human selection plays an important role in fruit flavor quality (Tieman et al., 2017; Klee and Tieman, 2018; Li et al., 2020). Fruit of wild melons have very thin light green flesh, are not sweet, are sometimes bitter and without aroma. In contrast, domesticated melon cultivars have larger fruit, non-bitter and thicker flesh, with considerably improved flavor (Pitrat, 2013). In terms of cultivated melons, postharvest handling and the retail system seem to be major contributors to poor fruit flavor. For example, heat treatment changes the aroma profiles of melons, causing a reduced “fresh” aroma as well as increased unpleasant “sulfurous” and “fermented” aroma (Yu et al., 2021). Additionally, total volatiles in melon juice are reduced significantly after 10°C storage for 20 d (Sun et al., 2022). In tomato, chilling results in loss of fruit flavor through impacting various flavor-associated VOCs (Zhang et al., 2016). Different fruit have different suites of VOCs, which contribute to their unique flavor. Thus, understanding how postharvest handling affects the flavor of different fruits is an important step to restore and maintain superior fruit flavor.

The aim of the present work is to study the effect of cold storage on melon fruit flavor quality and VOCs associated with human preference and to understand molecular mechanisms responsible for flavor loss in the chilled climacteric background of melon. Here, comprehensive metabolomic and transcriptomic analyses were performed in melon fruit. Altered flavor-associated VAEs in response to chilling and following a recovery period were associated with expression changes of some key pathway genes and multiple TFs, particularly ripening-related TFs.

## Materials and methods

### Melon materials

Melon ‘HT’ (Huai Tian No. 1, a climacteric cultivar of oriental melon, *Cucumis melon* var. *Makuwa* Makino) fruit were grown in a greenhouse on the Huaibei Normal University campus in Huaibei, China. Fruits were harvested at the ripe stage (30 days after pollination). Fruits of uniform size and without mechanical damage were selected, washed and dried at room temperature.

### Fruit treatment

Fruit treatment was performed as described by Zhang et al. (2016). Briefly, melon fruits were divided into three groups: (A) stored at 4°C for 7 d (without chilling injury), and then transferred to room temperature (~22°C) for 1 d; (B) held at 4°C for 8 d until use (without chilling injury); (C) harvested on day 8 (8 d after the fruits of first two groups). Three replicates of each group were performed, and each replicate has three fruits.

### Consumer preference test and analysis

A triangle test was performed according to the method described previously (Radovich et al., 2004). The test consisted of 58 untrained panelists, including twenty-eight males and thirty females, aged 20-45 years. Panelists were not trained but received instructions regarding the evaluation procedure. Panelists were presented with three samples of chilled (Group B) or unchilled (Group C) fruit, two were identical, and one was different. The samples were assigned blinding codes and randomized before testing. Panelists were instructed to identify the odd sample among the three by tasting all in the order presented. Panelists were provided with bottled water and sugar-free biscuits for palate cleansing, which they used between samples. Finally, they were asked to comment on the flavor and taste variation observed. The critical number of correct and incorrect responses for significance analysis was determined as described by Civille and Carr (2015). A binomial test was used to test for significance based on the correct number of accessors (Lawless and Heymann, 2010). The number of correct responses greater or equal to 27 of 60 panelists was considered a significant difference. Ambiguous responses were removed from the data. A plot of the frequency of the flavor descriptors for the chilled and unchilled fruit were obtained using the ggplot2 package in R (Wickham, 2016).

### Volatile analysis by gas chromatograpghy-ion mobility spectrometry

GC-IMS is a powerful technique for the separation and sensitive detection of VOCs and is widely used to analyze VOC changes during food storage (Wang et al., 2020). To investigate the volatile changes of HT1 melon cultivar during cold storage, the analysis of volatile fingerprints was performed on an HS-GC-IMS system (FlavourSpec^®^ Gesellschaft für Analytische Sensorsysteme mbH, Dortmund, Germany) as described by Yu et al. (2021). Three pooled fruit (three replicates for each group) were homogenized in a blender. For each replicate of melon sample, 10 g of melon juice was combined with 3 ml 200 mM ethylenediaminetetraacetic acid (EDTA) and 3 ml 20% CaCl_2_ (m/v) in a 20 ml headspace vial, the samples were stored at -80°C and mixed well before use. The GC system is equipped with an automatic headspace sampler unit (CTC Analytics AG, Switzerland), which can collect samples from the headspace vials with a heated air-tight syringe (1 ml). Next, the prepared ‘HT1’ samples in a 20 ml headspace vial were incubated at 40°C for 20 min with an agitation speed of 450 rpm, and 500 μl of headspace was injected into the GC injector at 80°C using a heated gas-tight syringe (45°C) with an injection speed of 30 ml min^-1^ and the GC program was as follow: 2 ml min^-1^ for 2 min, ramped up to 100 ml min^-1^ over 18 min, and held for 10 min. The separation was carried out on both a nonpolar FS-SE-54-CB capillary column (15 m × 0.53 mm × 0.25 μm, CS-Chromatographie Service GmbH, Durem, Germany) and a polar DB-Wax column (15 m × 0.53 mm × 0.25 μm, Agilent, Santa Clara, CA, USA) at isothermal condition of 60°C. Nitrogen was the gas at a flow rate of 150 ml min^-1^. The drift tube is 5 cm in length, and it was operated at a constant voltage of 400 V cm^-1^ at 45°C with a 150 ml min^-1^ N_2_ flow. Each IMS spectrum is an average of 12 scans. Each GC-IMS analysis was run in triplicate. To eliminate cross-contamination, the syringe was purged by an N_2_ flow for 30 s before every analysis and another 5 min after every analysis. n-ketones C_3_-C_9_ (Sinopharm Chemical Reagent Beijing Co., Ltd., China) were employed as external references to calculate the retention index (RI) of each compound. All volatile compounds were identified by comparing RI and normalized drift time (RIP relative) with the authentic reference compounds in the GC-IMS library. Analysis was performed on three replicates of each group.

### cDNA preparation, quantitative PCR, and sequencing

Total RNA from different treatments was extracted using TRIzol reagent (Life Technologies) and first-strand cDNAs were synthesized using the ReverTra Ace quantitative real-time PCR (qRT-PCR) Kit (Toyobo, Osaka, Japan) according to the manufacturer’s instructions. qRT-PCR was performed using the SuperReal PreMix Plus Kit (Tiangen, Beijing, China) in CFX Connect real-time PCR detection system (Bio-Rad, Hercules, CA, USA) according to the manufacturers’ instructions. Actin was used as the internal control (Primers used are listed in **Supplementary Table S13**). Total RNA samples were submitted for Next-Generation Sequencing (NGS) using Illumina NovaSeq (Shanghai Personal Biotechnology Co., Ltd, China). Briefly, the paired-end (PE) method was applied for RNA sequencing and the read length was 150 base pairs. The mapped reads for each sample ranged from 37,300,202 to 46,422,461 and the transcriptome coverage varied from 94.72% to 95.15%.

### RNA-Seq data analysis

The procedure of analysis was performed as described by (Guo et al., 2021). Briefly, the adapter sequence and low-quality value (QV < 20) from the raw reads in FastQ format were removed using Cutadapt (Version 1.1) software (Kim et al., 2015). The pruned clean reads were aligned to the melon reference genomic sequence Melon (DHL92) v3.5.1 Genome database (http://cucurbitgenomics.org/organism/18) using HISAT2 software (v. 2.1.0). HTSeq (v. 0.1.1) was used to calculate the read count as the original gene expression. The transcript abundance was indicated by normalized Fragments Per Kilo bases per Million fragments (FPKM), and genes with an average value higher than 1 were considered as expressed genes, while the genes expressed at very low levels were removed. Pearson correlation coefficient was estimated to examine the correlation of gene expression levels among samples. Based on principal component analysis (PCA) in “DESeq” package in R software, samples in each treatment group were clustered according to relative expression. Absolute log2 fold change (FC) > 1 and adjusted *p*-value < 0.05 were differentially expressed genes (DEGs) in each treatment. Expression levels of DEGs in each group were visualized as a heat map using “Pheatmap” package. The DEGs in each treatment group were further submitted for functional enrichment analysis of gene ontology (GO) and transcription factor (TF). For GO enrichment analyses performed in “TopGO” package, DEGs were divided into GO molecular function (MF), biological process (BP), and cellular component (CC). The TF information and relative family classification were predicted according to Plant Transcription Factor Database (PlantTFDB).

### Metabolite extraction and analysis

Metabolite profiling was performed using a non-targeted metabolome method by Personal Biotechnology Co. Ltd. (Shanghai, China). The materials used for the transcriptomic analysis were the same as those in metabolite profiling. Extraction and analysis of metabolites were performed as previously described (Sangster et al., 2006; De Vos et al., 2007). 200 mg ( ± 1%) of melon fruit was accurately weighed in an EP tube, and then 0.6 ml 2-chlorophenylalanine (4 ppm) methanol (-20°C) was added, followed by vortexing, grinding, and centrifugation. Finally, 20 μl from each sample was taken to the quality control (QC) samples (These QC samples were used to monitor deviations of the analytical results from these pool mixtures and compare them to the errors caused by the analytical instrument itself), and the samples were used for LC-MS detection.

Chromatographic separation was accomplished in a Vanquish system (Thermo, USA) equipped with an ACQUITY UPLC^®^ HSS T3 (150 mm x 2.1 mm, Waters, USA) column maintained at 40°C. The temperature of the autosampler was 8°C. Gradient elution of analytes was carried out with 0.1% formic acid in water (A2) and 0.1% formic acid in acetonitrile (B2) or 5 mM ammonium formate in water (A3) and acetonitrile (B3) at a flow rate of 0.25 ml min^-1^. Injection of 2 μl of each sample was done after equilibration. An increasing linear gradient of solvent B2/B3 (v/v) was used as follows: 0-1 min, 2% B2/B3; 1-9 min, 2%-50% B2/B3; 9-12 min, 50%-98% B2/B3; 12-13.5 min, 98% B2/B3; 13.5-14 min, 98%-2% B2/B3; 14-20 min, 2% B2-positive model (14-17 min, 2% B3-negative model). The ESI-MSn experiments were executed on the Thermo Q Exactive HF-X mass spectrometer with the spray voltage of 3.5 kV and -2.5 kV in positive and negative modes, respectively. Sheath gas and auxiliary gas were set at 30 and 10 arbitrary units, respectively. The capillary temperature was 325°C. The analyzer scanned over a mass range of m/z 81-1,000 for a full scan at a mass resolution of 60,000. Data dependent acquisition (DDA) MS/MS experiments were performed with HCD scan. The normalized collision energy was 30 eV. Dynamic exclusion was implemented to remove some unnecessary information in MS/MS spectra.

### Weighted gene co-expression network analysis

Co-expression networks were created using WGCNA (v1.29) package in R as described by (Langfelder and Horvath, 2008). The data used for WGCNA were performed on aroma components (alcohols, aldehydes, ketones, and esters) produced during storage. All genes and TFs were used as input to the signed WGCNA network construction. Modules were identified using “one-step network construction and module detection function” method with a soft thresholding power of 6 and a relatively large minimum module size of 30, and the threshold for merging modules was 0.25.

### Promoter analysis

Promoter sequences (base pairs -1,500 to -1) were obtained from Melon (DHL92) v3.5.1 Genome (http://cucurbitgenomics.org/organism/3). Transcription binding motifs were analyzed with plantCARE (http://bioinformatics.psb.ugent.be/webtools/plantcare/html/) and PlantPAN 3.0 (http://plantpan.itps.ncku.edu.tw/promoter.php) (Lescot et al., 2002; Li et al., 2020).

### Statistical analysis

Student *t*-test analysis was applied to compare the variation of volatile content and gene expression between Group A & B, B & C and A & C (Graphpad Prism 9). Letters and asterisks indicate significant differences (*p*-value < 0.05) for treatments at each measure time. PCA, DEGs, and GO enrich were described in each method section.

### Accession numbers

All accession numbers were obtained from the Melon (DHL92) v3.5.1 Genome database. *CmLOX18* (MELO3C024348.2), *CmADH1/2* (MELO3C023685.2/MELO3C014897.2), *CmHPL1/2* (MELO3C010910.2/MELO3C018413.2), *CmAAT1/3* (MELO3C024771.2/MELO3C024762.2), *CmMGL* (MELO3C013774.2), *CmPDC1/2* (MELO3C009145.2/MELO3C007227.2), *CmCXE1/2* (MELO3C010887.2/MELO3C014690.2), *CmBCAT1* (MELO3C010776.2), *CmArAT1* (MELO3C025613.2), *CmAADC1* (MELO3C008357.2), *CmACO1* (MELO3C014437.2), *CmACS1* (MELO3C021182.2), *CmNOR* (MELO3C016540.2), *CmMYB44* (MELO3C007586.2), *CmbZIP1* (MELO3C013408.2), *CmCBF1* (MELO3C006869.2).

## Results

### Chilling resulted in changes of melon VOC profiles and consumer liking

**Figure 1:**
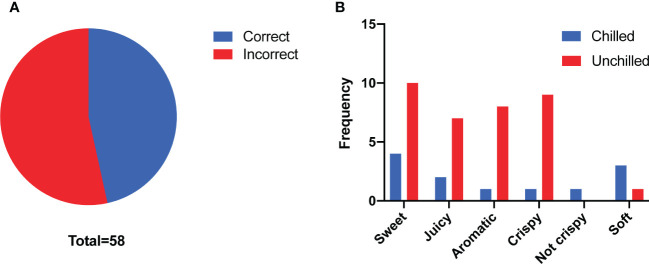
Consumer panel analyses. **(A)** Number of correct answers (n = 27) and incorrect answers (n = 31) from triangle test. **(B)** Taste descriptors for the chilled and unchilled melon fruit according to their frequency of use. Fewer panelists rated the chilled melon fruit as sweet, juicy and aromatic compared to the unchilled melon fruit.

To gain insight into how chilling affects flavor of melon fruit and consumer liking, we performed a triangle test to evaluate the flavor differences between chilled and unchilled fruit. Twenty-seven of fifty-eight panelists were able to identify significant differences in the flavor of the chilled compared to the unchilled (n > 26, p = 0.02 < 0.05) (**Figure 1A**). In addition, they described the unchilled melon as sweeter, juicier, and more aromatic than the chilled fruit, indicating that chilling had an adverse effect on flavor (**Figure 1B**).

**Figure 2:**
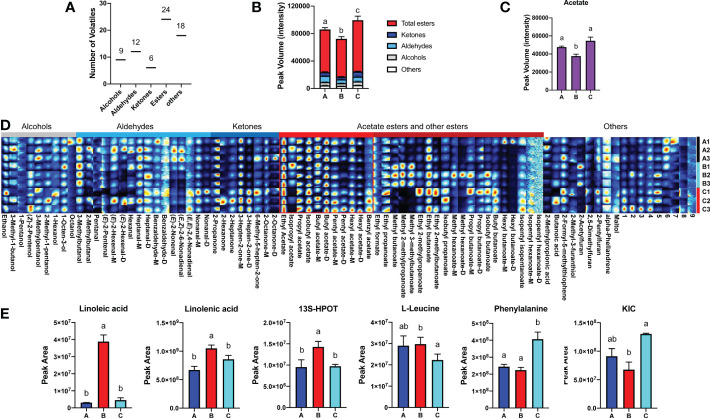
Changes of melon volatile content and metabolites in response to chilling. **(A)** Classification and numbers of all volatiles detected. **(B)** Statistical analysis of changes in VOC content after chilling ( ± SE, n = 3). **(C)** Chilling-induced changes in total VAE content ( ± SE, n = 3). **(D)** Fingerprint of all volatiles detected in three groups. Each row represents all signal peaks in each sample, and each column represents the signal peak of the same VOC in different samples. The intensity of the ion signal is described by different colors, red indicates high intensity. VOC names are listed below each column, and some volatiles are followed by M and D, which are the monomer and dimer of the same volatile. The numbers indicate unidentified VOC peaks. Information of all volatiles is listed in **Supplementary Table S1**. **(E)** Statistical analysis of metabolites in fatty acid and amino acid pathways after chilling ( ± SE, n = 6). Significant differences in volatiles and metabolites are denoted by different letters (*p*-value < 0.05). HPOT, Hydroperoxy octadecatrienoic acid; KIC, Ketoisocaprorate. Group A, stored at 4°C for 7 d, and then transferred to room temperature (~22°C) for 1 d; Group B, held at 4°C for 8 d; Group C, melon fruit were harvested 8 d after the fruit of first two groups.

Subsequently, VOCs extracted from melon fruit after 7 d chilling followed by 1-d recovery at room temperature (Group A), after 8 d chilling only (Group B), and harvested on day 8 (Group C) were quantified. Sixty known and nine unknown VOCs were detected in fruit from all three groups and used for further analysis (**Supplementary Table S1**). These VOCs consist of nine alcohols, twelve aldehydes, six ketones, twenty-four esters, and eighteen others (**Figure 2A**). Among the VOCs, esters are the most abundant (> 70% of total VOC content) in all groups, and ~70% of the esters are VAEs. Based on the VOC content, melon fruit from the three different groups were separated by PCA, and biological replicates clustered together (**Supplementary Figure S1**). 2D ion mobility spectrums of volatiles in fruit from these three groups were shown (**Supplementary Figure S2**). In general, the concentration of volatiles in 8-d chilled fruit (Group B) was lower in comparison with unchilled fruit (Group C). Total amount of volatiles in 8-d chilled fruit decreased by ~73% relative to the unchilled. After 1-d recovery at room temperature (Group A), total VOC content increased, but was still lower compared to the unchilled (**Figure 2B**). In particular, content of VAEs was lower after chilling and recovery at room temperature for 1 d. However, total content of volatile alcohols or aldehydes was not substantially affected after cold storage compared to the unchilled (**Figures 2B, C**). Additionally, no significant differences were observed in fructose, glucose and citrate between the chilled and the unchilled fruit (**Supplementary Figure S3**).

A gallery plot was performed to better visualize major chilling-induced changes to VOC profiles. A total of eighty-two detected signal peaks were characterized (**Figure 2D**). Although total content of alcohols or aldehydes was not altered after cold storage compared to the unchilled fruit, content of several alcohols and aldehydes was lower, including 3-methylpentanol, nonanal and (*E*, *E*)-2,4-nonadienal. In addition, total ketone content was also reduced in 8-d chilled fruit. Among the ketones, content of 3-hepten-2-one and 2-propanone was lower. Total content of VAEs in the chilled fruit was ~30% lower compared to that in unchilled fruit and those recovered after transfer to the room temperature. Content of multiple VAEs was reduced after cold storage, including propyl acetate, isobutyl acetate, pentyl acetate, hexyl acetate, and benzyl acetate. Chilling-induced decreases were also observed for other esters, including isopentyl isopentanoate, butyl butanoate, isobutyl butanoate, and isopentyl hexanoate. Content of the aforementioned VAEs increased after 1-d recovery. However, some VOCs were found to increase after cold storage compared to the unchilled. Interestingly, these VOCs mainly included methyl esters, such as methyl butanoate, methyl hexanoate, methyl 3-methylbutanoate, and methyl 2-methylpropanoate (**Supplementary Table S1**). In addition, content of these methyl esters was reduced after return to room temperature. These results indicate that chilling affects the flavor of melon fruit, and chilling-induced changes to melon VOC profiles are mainly involved in flavor-associated esters, particularly VAEs.

### Metabolomic analysis on the VAE-related pathways in response to chilling

The aforementioned VAEs are derived from fatty acid and amino acid pathways. In response to chilling, metabolic flux throughout these two pathways is critical for downstream VAE biosynthesis. Therefore, multiple intermediate metabolites were investigated, including linoleic acid, linolenic acid, 9(*S*)-hydroperoxy-10(*E*),12(*Z*),15(*Z*)-octadecatrienoic acid (9(*S*)-HPOT), and 13(*S*)-hydroperoxy-9(*Z*),11(*E*),15(*Z*)-octadecatrienoic acid (13(*S*)-HPOT) from the fatty acid pathway, in addition to isoleucine, L-leucine, valine, phenylalanine, 3-Methyl-2-oxovaleric acid and Ketoisocaprorate (KIC) from the amino acid pathway. According to the analysis of differentially accumulated metabolites, we found that content of linoleic acid, linolenic acid, and 13*S*-HPOT increased after chilling and was reduced after 1-d recovery at room temperature (**Figure 2E**; **Supplementary Table S2**). However, content of metabolites from the amino acid pathway showed inconsistent patterns in response to chilling. Content of L-leucine was higher in the chilled fruit than the unchilled and remained high after cold storage followed by 1-d recovery. Content of KIC, a ketoacid generated from L-leucine, was reduced after chilling and slightly increased after recovery. Phenylalanine content was lower after cold storage and remained at a low level after 1 d returning to room temperature (**Figure 2E**; **Supplementary Table S2**). These results suggested that chilling affects VAE biosynthesis *via* regulation of metabolic flux.

### Transcriptomic response to cold storage

**Figure 3:**
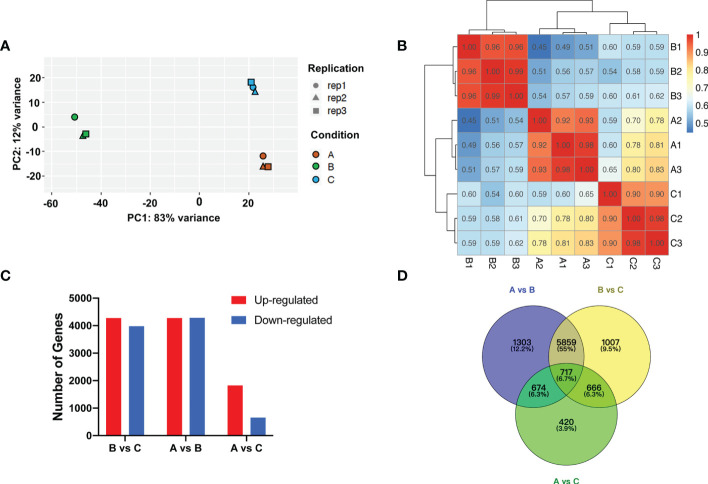
Transcriptomic analysis after cold storage. **(A)** Principal component analysis of differential expressed genes. Different symbols and colors represent different replicates and treatment conditions, respectively. **(B)** Correlation analysis of patterns of RNA-Seq in each group. **(C)** Number of up- and downregulated genes among groups. **(D)** Venn diagram of number of DEGs among groups.

RNA-Seq data was generated for transcriptomic analysis. After discarding adaptor and low-quality reads, ~6.2 Gb clean reads (>90% of total reads) on average were produced (**Supplementary Table S3**). Principle components analysis (PCA) of RNA-Seq data revealed the relationship between experimental samples, which exhibited clear separation for the samples of the aforementioned three groups (**Figure 3A**). Pearson correlation coefficient analysis indicated that, in general, replicates after chilling followed by 1-d recovery at room temperature (Group A) and harvested on day 8 (Group C) were less correlated with the replicates after chilling (Group B) (**Figure 3B**).

Analysis of Group A & B, and Group B & C identified 8553 (4272 upregulated and 4281 downregulated) and 8249 (4272 upregulated and 3977 downregulated) DEGs, respectively. However, only 1824 upregulated and 653 downregulated DEGs were identified *via* analysis of Group A and C (**Figures 3C, D**). Based on the expression levels of genes from each group, correlation among the samples was calculated and hierarchical clustering was visualized in a heatmap. Consistent with PCA analysis, gene expression clusters in Group B were distinguishable from that in Group A and Group C (**Supplementary Figure S4**). These results indicate that expression levels of many genes are sensitive to temperature shift. The C-repeat binding factor (CBF)/dehydration responsive-element binding (DREB) TFs are known to be sensitive to low temperature (Thomashow, 2010; Zhang et al., 2016). In the present study, we found that transcript levels of multiple melon CBFs/DREBs were higher in the chilled melon and returned to unchilled levels after transfer to room temperature (**Supplementary Figure S5**).

### Responses of VAE biosynthetic genes to chilling

**Figure 4:**
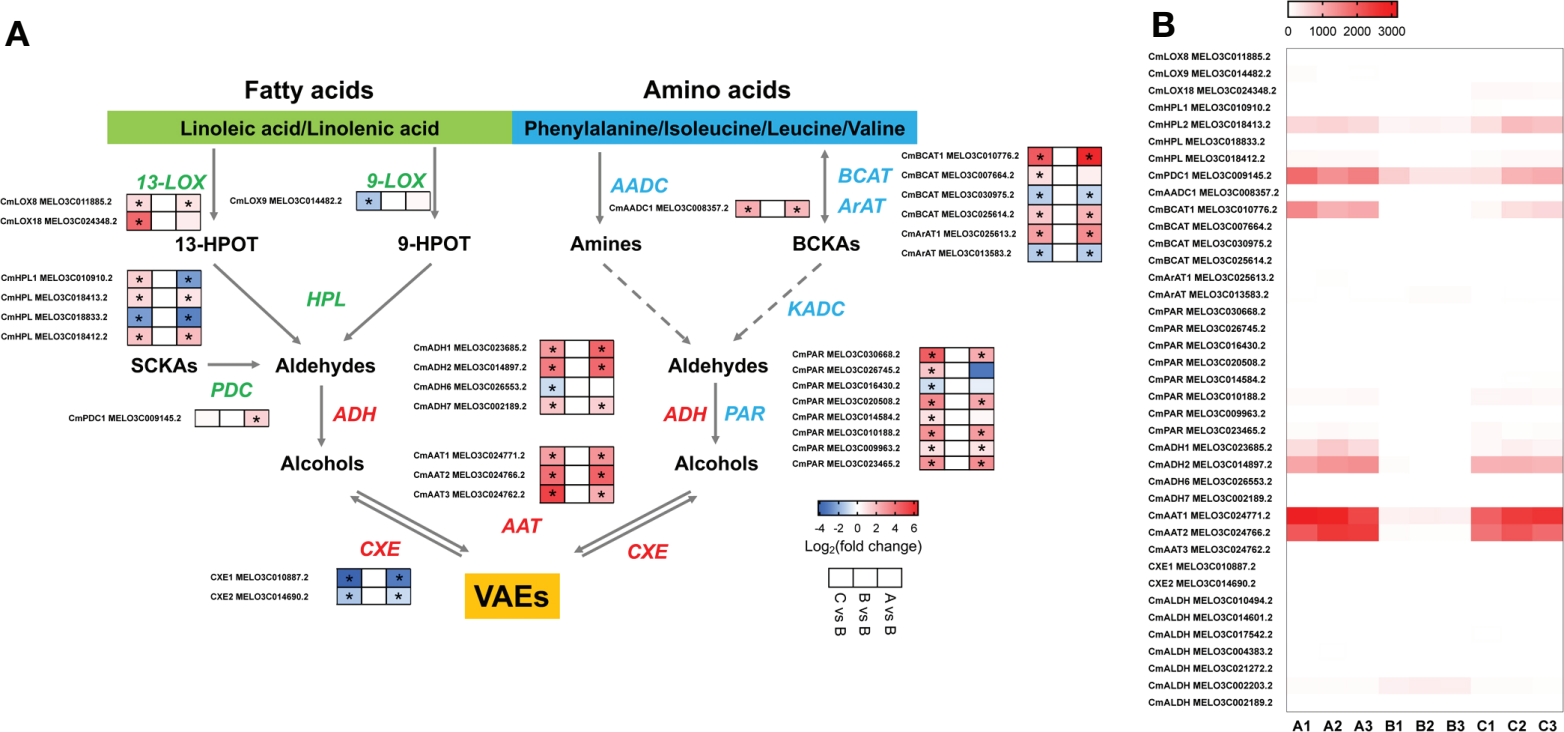
The analysis of differentially expressed genes in VAE biosynthesis pathway. **(A)** VAE biosynthesis in fatty acid and amino acid pathways. **(B)** A heatmap of DEGs involved in these pathways. Values of log2(foldchange) and FPKM are used in the heatmap of A and B, respectively. 13-HPOT, 13(*S*)-hydroperoxy-9(*Z*),11(*E*),15(*Z*)-octadecatrienoic acid; 9-HPOT, 9(*S*)-hydroperoxy-10(*E*),12(*Z*),15(*Z*)-octadecatrienoic acid; SCKAs, straight-chain ketoacids; BCKAs, branched-chain ketoacids; LOX, lipoxygenase; HPL, hydroperoxide lyase; PDC, pyruvate decarboxylase; BCAT, branched chain amino transferase; ArAT, aromatic amino transferase; AADC, amino acid decarboxylase; ADH, alcohol dehydrogenase; PAR, phenylacetaldehyde reductase; AAT, alcohol acyltransferase; CXE, carboxylesterase; ALDH, aldehyde dehydrogenase; KADC, ketoacid decarboxylase; VAEs, volatile acetate esters. Asterisks indicate significant differences (*p*-value < 0.05).

To understand the molecular basis of the reduction of melon flavor-associated VAEs during cold storage, we investigated the relevant biosynthetic pathways and genes involved in the biosynthesis of VAEs. As observed in the aromatic melon fruit that we used, content of volatile esters was much higher than other characterized VOCs, such as alcohols and aldehydes (**Figure 2B**; **Supplementary Table S1**). Alcohol dehydrogenase (ADH) and alcohol acyltransferase (AAT) are able to convert volatile aldehydes to their corresponding alcohols and esters (**Figure 4A**). The expression levels of two ADH family members (*CmADH1* and *CmADH2*) were downregulated after 8-d cold storage and upregulated to higher levels after 1-d recovery at room temperature, and the transcript levels after recovery were higher than day 8. Similarly, the transcript abundance of two members of AAT family (*CmAAT1* and *CmAAT3*) was lower in the chilled melon and recovered after 1 d at room temperature (**Figure 4B**; **Supplementary Tables S4**, **S5**).

In the fatty acid pathway, biosynthesis of C5 and C6 green leafy volatiles depends upon LOX and HPL activities (**Figure 4A**). Here, we observed *CmLOX18* expression level was lower after chilling and recovered after 1 d at room temperature. In addition, expression levels of *CmLOX8* and a 9-lipoxygenase (*CmLOX9*) were also altered after chilling. Transcript levels of two CmHPLs were lower in the chilled melon. *CmHPL2* expression was recovered after return to the room temperature, while *CmHPL1* remained at low level. CmPDC1 involved in aldehyde biosynthesis through catalyzing straight-chain ketoacids was reduced after chilling and recovered to higher level after 1 d at room temperature (**Figure 4B**; **Supplementary Tables S4**, **S5**). In the amino acid pathway, *CmBCAT1* and *CmArAT1* expression levels were reduced in the chilled melon fruit and upregulated after 1 d of recovery. Moreover, multiple aldehyde dehydrogenases (ALDHs) were also sensitive to low temperatures (**Figure 4B**; **Supplementary Tables S4**, **S5**). Therefore, reduced production of VAEs after chilling cannot be directly explained by a single enzyme activity alone.

### Chilling-induced expression variations in transcription factors and hormone regulators

According to the expression patterns of DEGs among the three groups, clustering analysis was carried out, and DEGs were divided into nine clusters (**Supplementary Figure S6**). Based on the Plant/Animal Transcription Factor Database (Plant/AnimalTFDB), thirty TF families were identified (**Supplementary Figure S7**; **Supplementary Table S6**). To narrow the scope of differentially expressed TFs, we removed the genes without significance (*p*-value > 0.01), and 375 TFs were selected. 246 TFs belong to Cluster 1 and 5, indicating that their expression levels were higher after chilling and recovered after 1 d at room temperature. On the contrary, 129 TFs were clustered in Cluster 6 and 8, indicating that their expression levels were lower after chilling and increased after 1-d recovery (**Supplementary Table S7**).

Clusters 6 and 8 identified melon *NONRIPENING* (*CmNOR*) which is a key ripening regulator, and its expression was reduced (~30-fold reduction) after chilling and upregulated (~40-fold increase) after 1-d recovery at room temperature, which is consistent with the pattern of ester levels and flavor intensity (**Supplementary Tables S4**, **S5**). In the same cluster with *CmNOR*, *CmMYB44*, an ester-associated apple MdMYB6 homolog, had lower expression during cold storage and increased transcript after 1-d recovery (**Supplementary Tables S4**, **S5**). In addition, Cluster 1 and 5 identified a bZIP family gene (named *CmbZIP1*, ester-associated MabZIP4 homolog in banana) with the highest expression during chilling and reduced transcript levels after recovery (**Supplementary Tables S4**, **S5**).

Ethylene also regulates various aspects of ripening, including VOC biosynthesis in fruit. Ethylene biosynthesis genes, including aminocyclopropane carboxylate synthase (ACS) and oxidase (ACO), were investigated. The transcript levels of *CmACS1* and *CmACO1* were the highest among the family members, respectively. The expression of *CmACO1* was lower in the chilled melon and increased after 1 d at room temperature. Conversely, much higher transcript levels of *CmACS1* was observed after cold storage and declined after returning to room temperature (**Supplementary Figure S8**; **Supplementary Tables S4**, **S5**).

### WGCNA identified the aforementioned ester-associated genes in response to temperature changes

**Figure 5:**
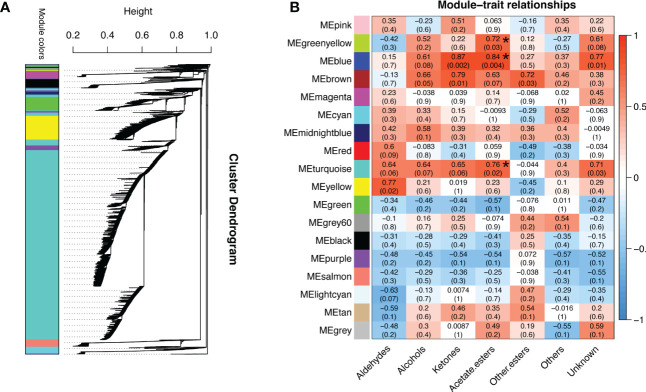
Analysis of gene co-expression and the relationships between genes and volatile content in response to cold storage. **(A)** Hierarchical cluster tree exhibits 18 modules of co-expressed genes. Each branch represents one gene in the tree. **(B)** Module-trait correlations and gene co-expression network analysis performed by weighted gene co-expression network analysis during cold storage. The green-yellow, blue and turquoise modules with asterisks significantly related to the content of volatile acetate esters. The corresponding *p*-values are shown in parentheses. The left to right panel represents the module-trait correlations from -1 to 1.

**Figure 6:**
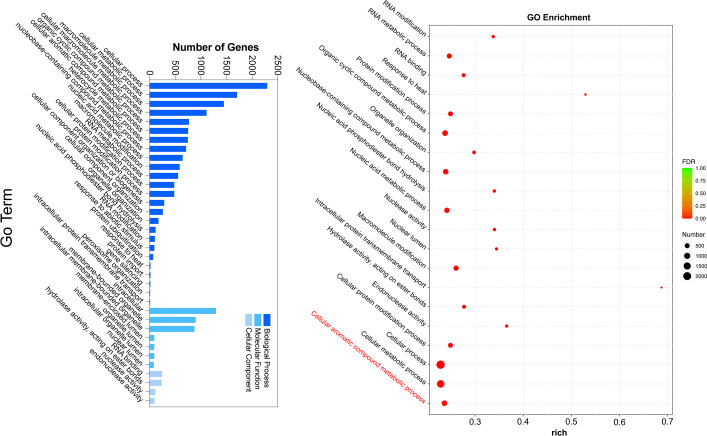
GO functional enrichment analysis of DEGs in chilled and unchilled melon fruit.

To gain further insight into the regulation of ester biosynthesis after temperature shift, WGCNA was performed to investigate the co-expression networks of key DEGs. According to the similarity of expression patterns, genes were grouped into a total of eighteen co-expression modules (**Figure 5A**). Based on the top three coefficient values shown in the heatmap of module-trait correlations, content of esters was positively correlated with gene expressions in the blue, turquoise and green-yellow modules (**Figure 5B**; **Supplementary Table S8**). Subsequently, GO annotation analysis was performed to summarize functions of the DEGs identified in the modules, and DEGs in response to chilling were investigated. The functional categories of the DEGs included biological process, molecular function and cellular component. Top twenty GO terms indicated that cellular process in the biological process ranked the highest, and 753 DEGs were divided into the group of cellular aromatic compound metabolic process (**Figure 6**; **Supplementary Table S9**).

**Figure 7:**
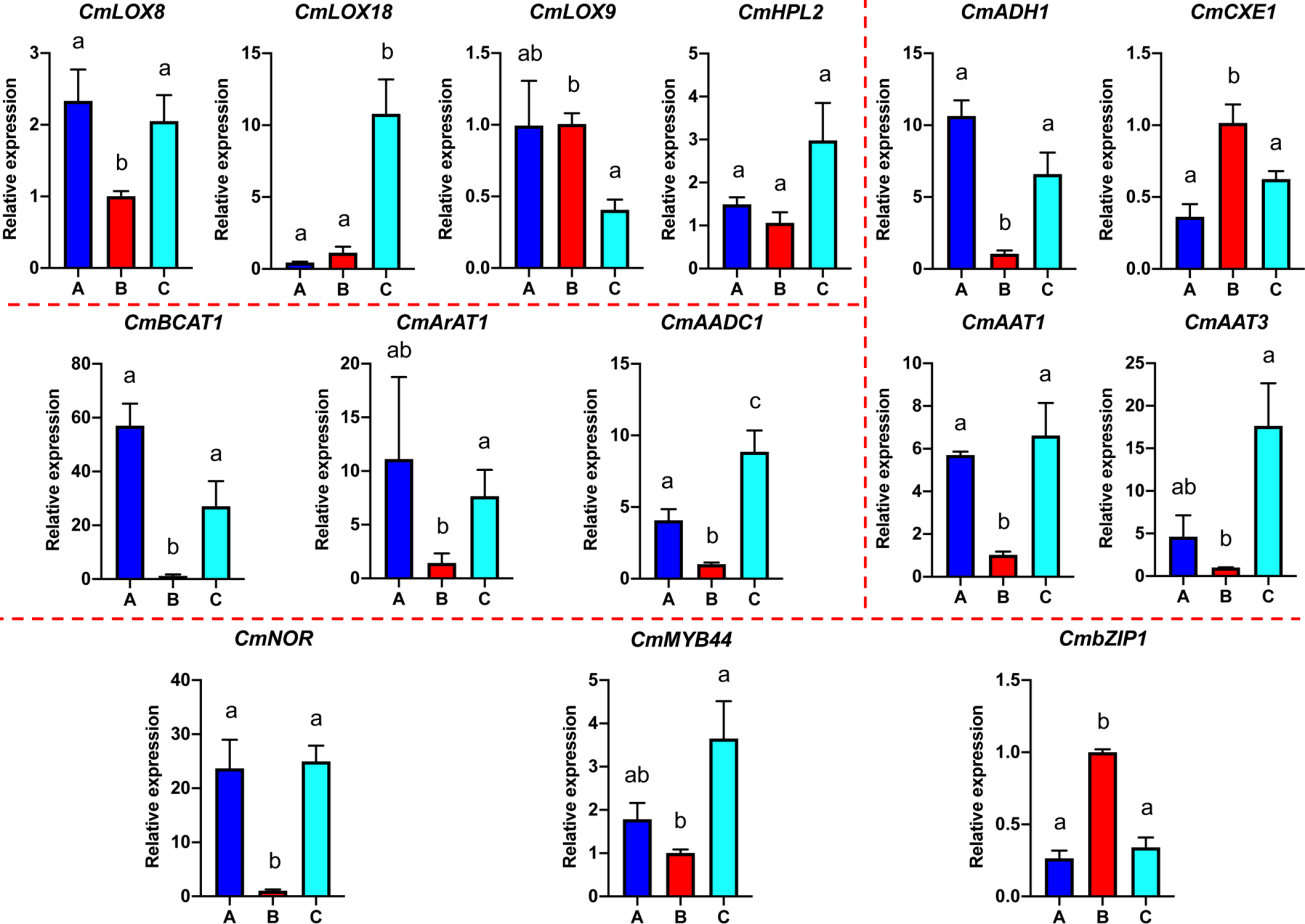
Quantitative real-time PCR analysis of select VAE-related DEGs. Significant differences in gene expression are denoted by different letters ( ± SE, n = 3, *p*-value < 0.05).

Based on the analyses of transcriptome and metabolome, multiple genes involved in ester biosynthesis were preliminarily identified. Notably, WGCNA further narrowed the scope of DEGs correlated with ester biosynthesis. Two lipoxygenases (*CmLOX8* and *CmLOX18*), one hydroperoxide lyase (*CmHPL2*), one alcohol acyltransferase (*CmAAT1*), one carboxylesterase (*CmCXE1*), and one amino acid decarboxylase (*CmAADC1*) were identified. These DEGs were confirmed by qRT-PCR using RNA from fruit after chilling followed by 1-d recovery at room temperature (Group A), chilled (Group B) and unchilled fruit (Group C) (**Figure 7**). Additionally, WGCNA was also performed to investigate the correlation between differentially expressed TFs and ester content during chilling and 1-d recovery after chilling. Seventeen key TFs (GS > 0.85, GS < -0.85) were identified, including twelve positive regulators (one C3H, one CPP, one ERF, two GATAs, one GeBP, one LBD, one MYB, one STAT and three WRKYs) and five negative regulators (one ARR-B, two bHLHs, one DBB and one MYB-related) (**Table 1**). These positive regulators exhibited lower expression levels in the chilled fruit and were upregulated after 1-d recovery at room temperature, which was consistent with the pattern of altered ester content. However, the negative regulators exhibited higher levels after chilling and were downregulated after 1-d recovery. Although the aforementioned three TFs, *CmNOR*, *CmMYB44*, and *CmbZIP1* were not included in the list of top TFs, WGCNA revealed that the relative GS values were higher than 0.7 or lower than -0.7, indicating significant correlations between expression and ester content (**Supplementary Table S10**).

**Table 1:** The candidate transcription factors potentially involved in VAE biosynthesis.

TF family	Gene ID	Description	Correlation with VAEs	*p*-value
ARR-B	MELO3C016975.2	two-component response regulator ARR12-like	-0.9043733	0.00080999
bHLH	MELO3C023532.2	transcription factor bHLH130-like isoform X2	-0.8627904	0.00274777
	MELO3C020781.2	transcription factor bHLH121 isoform X1	-0.8782129	0.00183893
C3H	MELO3C015665.2	zinc finger CCCH domain-containing protein 14-like	0.86642181	0.00251085
CPP	MELO3C006510.2	Protein tesmin/TSO1-like CXC 2	0.86381782	0.00267924
DBB	MELO3C016115.2	B-box zinc finger protein 22	-0.8500681	0.00369915
ERF	MELO3C014181.2	ethylene-responsive transcription factor 3	0.85900818	0.00301046
GATA	MELO3C011079.2	GATA transcription factor 25	0.87782345	0.00185886
	MELO3C007882.2	GATA transcription factor	0.87543688	0.00198426
GeBP	MELO3C022945.2	mediator-associated protein 1-like	0.86541279	0.0025752
LBD	MELO3C005013.2	Lob domain-containing protein 1	0.87612233	0.00194767
MYB	MELO3C012077.2	Myb family transcription factor family protein	0.93648017	0.0001998
MYB-Related	MELO3C020620.2	telomere repeat-binding protein 5-like	-0.9271353	0.00032002
STAT	MELO3C016678.2	SH2 domain protein B	0.85480225	0.00332233
WRKY	MELO3C016966.2	WRKY transcription factor	0.90052453	0.00092635
	MELO3C018717.2	WRKY protein	0.86917051	0.00234117
	MELO3C014066.2	WRKY transcription factor	0.85615109	0.00322

### Analysis of potential transcriptional binding motifs in *CmAAT1* and *CmAAT3* promoter sequence

Under chilling conditions, volatile aldehydes and alcohols were not substantially affected, and the last step of ester biosynthesis catalyzed by AAT seems to be an important control point. *cis*-regulatory elements in promoter regions containing TF binding sites play a critical role in regulation of gene expressions. Thus, 1.5-kb putative promoter sequences of *CmAAT1* and *CmAAT3* were investigated.

The analysis of *CmAAT1* and *CmAAT3* promoter regions identified motifs mainly involved in response to stress and hormone levels. For *CmAAT1*, stress responses included anaerobic induction (ARE), stress (STRE), wounding (WUN-motif), and hormone response consists of ethylene (ERE), MeJA (MYC and TGACG-motif) and salicylic acid (TCA-element). For *CmAAT3*, anaerobic induction (ARE), light (AE-box), wounding (WRE3) and ethylene (ERE), gibberellin (TATC-box), MeJA (MYC), salicylic acid (TCA-element) were found to be involved in stress and hormone responsiveness, respectively. In addition, various conserved motifs were found in the promoter region of *CmAAT1*, including G-box (bZIP binding site), MYB and ‘ACCGAC’ (DREB/CBF) and ‘TTA/GCGT’ (NAC). MBSI (MYB) and ‘TTA/GCGT’ (NAC) were found in the *CmAAT3* promoter region.

**Figure 8:**
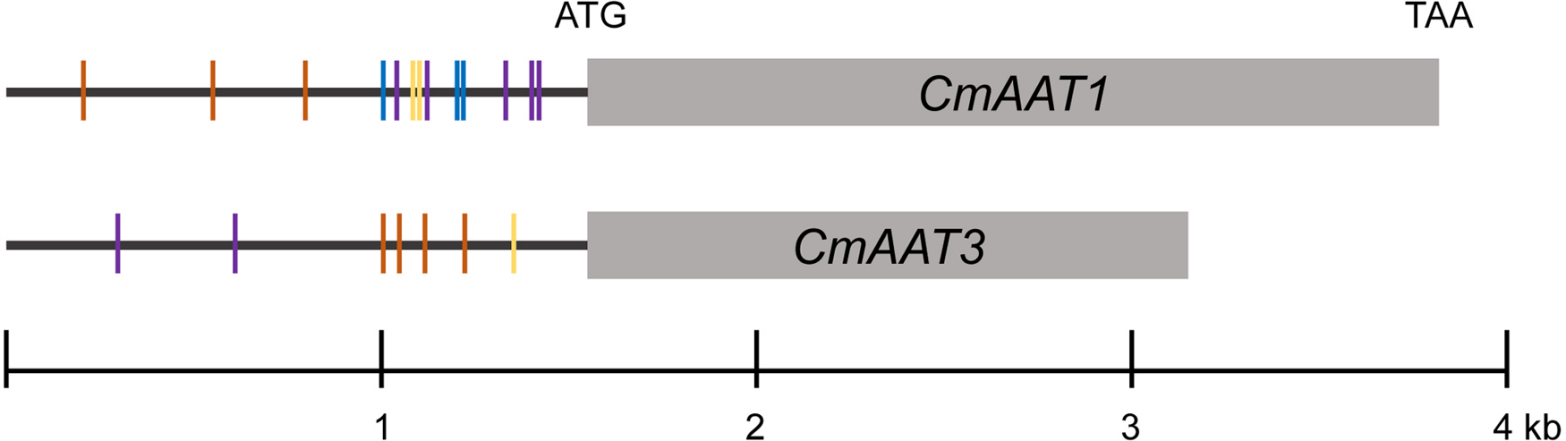
Analysis of *CmAAT1* and *CmAAT3* promoter sequences. TF binding motifs were shown in the 1.5-kb promoter regions. Purple, NAC (TTA/GCGT, (Lindemose et al., 2014); Blue, AP2/ERF (ACCGAC, Suzuki et al., 2005); Yellow, MYB (AACTG, Prouse and Campbell, 2012); Orange, AP2/RAV (CAACA, Zhang et al., 2020).

After temperature shift, we found that expression levels of many TFs exhibited the same pattern with the *CmAAT1* and *CmAAT3*. These TFs included Dof, ERF, WRKY and TCP, in addition to the aforementioned bZIP, MYB, MYC and NAC (**Table 1**; **Supplementary Tables S4**, **S5**). The putative binding sites of some selected TFs were also found in the 1.5-kb promoter sequence of *CmAAT1* and *CmAAT3* (**Figure 8**), and all putative motifs were shown in **Supplementary Table S11** and **S12**. These observations indicated potential roles of the aforementioned TFs in regulation of VAE-related *AAT* expression during cold storage.

## Discussion

For decades, consumers have complained about the flavor of commercial fruit. In addition to breeding of modern cultivars with poor flavor, postharvest chilling treatment is known to result in fruit flavor loss (Zhang et al., 2016; Tieman et al., 2017; Klee and Tieman, 2018). VOCs are critical contributors to fruit flavor quality. Although different fruit often share many aroma characteristics, each fruit has a distinctive aroma determined by the proportions of key volatiles as well as the presence or absence of unique components (Gonda et al., 2010). Therefore, chilling-induced changes to flavor-associated VOCs can be various among fruit species. In our present study, we found that chilling resulted in reduction of flavor-associated esters, particularly VAEs in melon fruit, while other VOCs were only slightly altered. Finally, comparative metabolomic and transcriptomic analyses during temperature shift from 4°C to room temperature were performed to uncover the causes of melon flavor deterioration after chilling.

In climacteric melon, fatty acid- and amino acid-derived esters are the most abundant as well as the most important contributors to fruit flavor (Gonda et al., 2016). Approximately 50% of VOCs are VAEs at a ripe stage in the climacteric melon fruit used in this study. A previous study revealed that content of VAEs was reduced at the later maturity of the oriental melon (Guo et al., 2017). As known, low temperature can slow ripening progress (Zhang et al., 2021). Here, we found that total content of VAEs was remarkably lower in the chilled ripe melon than the unchilled fruit. Thus, chilling-induced reduction of VAE content is more likely caused by transcriptional regulation of the metabolic pathways.

Multiple flavor-associated pathway genes have been identified, and each of them plays an important role in regulating flux of metabolites throughout respective pathways (2012; Speirs et al., 1998; Chen et al., 2004; Gonda et al., 2010; Goulet et al., 2012; Goulet et al., 2015; Wang et al., 2019). However, flavor-associated VOCs and molecular regulation in response to chilling are still not fully understood in many fruit crops such as melon. In addition to the well-known pathway genes, we found that additional genes involved in flavor-associated VOC synthesis were also sensitive to chilling. An aromatic amino acid decarboxylase gene (named *CmAADC1*) was expressed in a lower level during chilling and partially recovered after 1 d at room temperature. Its homolog, tomato LeAADC2 plays a critical role in the initial step of biosynthesis of 2-phenylethanol and 2-phenylacetaldehyde (Tieman et al., 2006). Additionally, a methionine-gamma-lyase gene (*CmMGL*) associated with the presence of sulfur volatiles in melon was upregulated during cold storage and returned to a considerably lower level after transfer to room temperature (**Supplementary Tables S4**, **S5**). GC-IMS analysis identified only sixty known VOCs and nine unknown VOCs (**Supplementary Table S1**). However, multiple VOCs that contribute to the fruit flavor were not measured in the used melon fruit, including 2-phenylethanol and sulfur volatiles. Sulfur volatiles are important contributors to melon aroma and can be detected in many melon varieties, but high levels may cause undesirable aroma (Gonda et al., 2013). Thus, it will be interesting to determine whether these flavor-associated VOCs are altered during temperature shift in melon fruit, other than VAEs.

In addition, expression levels of multiple TFs were altered in response to low temperature. In particular, some associated with fruit development were downregulated, including ripening-associated NOR and ethylene-responsive ERF (**Supplementary Tables S4**, **S5**). Altered expression of these TFs during temperature shift would be expected to change numerous ripening processes, permitting the organ to redirect metabolic resources into more suitable stress responses (Zhang et al., 2016). Additionally, ripening has been a major focus of plant breeding in fleshy fruits, with special effort on the improvement of organoleptic quality and post-harvest durability (Ríos et al., 2017). Ripening-associated ETHQV6.3 encoded by a NAC domain TF (CmNOR) advances the ethylene production and activates the production of esters, changing the aroma profile of melon fruit (Santo Domingo et al., 2022). Thus, the altered expression levels of ripening-associated TFs cannot be ignored when examining melon flavor loss after cold storage. Besides, TFs are able to regulate expression of downstream genes *via* binding to elements in the promoter region (Schwechheimer et al., 1998). Notably, NAC and ERF binding motifs were observed in the promoter sequences of ester-associated *CmAAT1* and *CmAAT3* (**Figure 8**). Motifs involved in stress and hormone responsiveness were also found in the aforementioned promoter regions.

Climacteric ripening is characterized by the autocatalytic biosynthesis of plant hormone ethylene (Ríos et al., 2017). Ethylene synthesis was reduced after chilling with partial recovery after transfer to room temperature in tomato (Zhang et al., 2016). In melon, low temperature treatments inhibited ethylene production during storage (Zhang et al., 2015). Ethylene biosynthesis genes included aminocyclopropane carboxylate synthase (ACS) and oxidase (ACO). Conversion of S-adenosylmethionine to 1-aminocyclopropane-1-carboxylic acid by ACS is the first and committal step in the ethylene biosynthesis pathway, and ACO is involved in the final step of ethylene production in plant tissues (Pech et al., 2008). Of the multiple ACS and ACO enzymes reported in the melon genome, *CmACS1* and *CmACO1* are specific to fruit ripening as their expression increases in climacteric fruit after the burst of ethylene (Miki et al., 1995; Saladié et al., 2015). Initially, it was postulated that not ACO, but ACS is the rate-limiting enzyme in ethylene biosynthesis pathway (Adams and Yang, 1979; Houben and Van de Poel, 2019). However, an increasing amount of evidence has been gathered over the years, which demonstrates the importance of ACO, and not ACS, in controlling ethylene production in plants (English et al., 1995; Vriezen et al., 1999; Shi et al., 2006; Love et al., 2009; Van de Poel et al., 2012; Chen et al., 2016). In our study, *CmACO1* expression was downregulated after chilling. However, *CmACS1* displayed an opposite expression pattern in response to low temperature. The last step of ethylene biosynthesis is likely to be controlled by rate-limiting enzyme CmACO1, rather than CmACS1 involved in the initial step.

Sugar and organic acid accumulation were ethylene-independent (Pech et al., 2008; Ríos et al., 2017). Accordingly, fructose, glucose and citrate were not substantially affected after chilling (**Figure S3**). Conversely, the production of aroma volatiles was strictly ethylene-dependent. Ethylene has an important role in ester biosynthesis in climacteric fruit including peach and apple (Wang et al., 2018; Cao et al., 2021; Santo Domingo et al., 2022). A previous study revealed that ethylene levels were lower in chilled oriental melon (Zhang et al., 2015), showing the same pattern as we observed for VAEs. Climacteric melon has a peak of ripening, controlled by increased ethylene (Pech et al., 2008; Santo Domingo et al., 2022). Multiple genes involved in ester biosynthesis were regulated by ethylene production in melon. The expression of *CmAAT1* was severely reduced in ethylene-suppressed antisense 1-aminocyclopropane-1-carboxylic acid oxidase fruit and in wild-type fruit treated with the ethylene antagonist 1-methylcyclopropene (1-MCP) (Yahyaoui et al., 2002). In addition, both 1-MCP and low temperature downregulated *CmLOX18* expression (Zhang et al., 2015). As observed in our work, a key ethylene regulator, *CmACO1* was downregulated after cold storage, which likely resulted in reduced ethylene production. Consistently, expression of *CmLOX18*, *CmAAT1* and *CmAAT3* were found to be decreased after chilling and recovered after a return to room temperature.

VAEs were remarkably altered in response to chilling. However, total volatile aldehydes and alcohols derived from fatty acids and amino acids were only slightly affected by chilling. During cold storage, the reduced *CmAAT1* and *CmAAT3* expression can be responsible for the lower VAE content, in turn resulting in higher volatile alcohols. Similarly, reduced transcript of *CmADH1* and *CmADH2* resulted in less conversion of volatile aldehydes. GC-IMS analysis showed that content of some aldehydes even increased after chilling. In addition, CXEs are responsible for conversion of VAEs to alcohols (Goulet et al., 2015). Interestingly, the transcripts of two tomato homologs in melon, *CmCXE1* and *CmCXE2*, were higher in chilled melon fruit and downregulated after 1-d recovery at room temperature (**Supplementary Tables S4**, **S5**), which indicated that CXE-catalyzed biosynthesis of volatile alcohols might increase during cold storage. Additionally, content of some upstream metabolites in the fatty acid pathway increased after cold storage. Thus, a metabolic balance of intermediate products occurred during cold storage, and it seems that chilling reduced VAE content was a result of transcriptional regulation.

## Conclusion

We combined sensory evaluation, transcriptomic and metabolomic analyses to determine the impact of postharvest chilling on the flavor of melon fruit. In summary, consumer panel tests indicated a negative change in the flavor of the chilled melon fruit in comparison with the unchilled. Melon flavor-associated VAEs derived from fatty acids and amino acids represents the dominant esters, showing considerably lower content after cold storage. Additionally, metabolomic analysis suggested that the corresponding upstream metabolites including linoleic acid, linolenic acid, phenylalanine and KIC were also altered in the chilled fruit. Transcripts for various key VAE synthesis genes, such as *CmLOX18*, *CmADH1*, *CmAAT1*, *CmCXE1*, *CmArAT1* and *CmBCAT1*, ripening-related TF, such as *CmNOR* and hormone regulators, such as *CmACO1* were significantly changed during cold storage. Most of the chilling-induced changes recovered after transfer to the room temperature. In general, chilling resulted in flavor loss of melon fruit, in large part due to the reduced VAE content. Our study provided a molecular basis for the understanding of melon fruit flavor loss during cold storage.

## Data availability statement

The RNASeq data presented in the study are deposited in the NCBI-GEO (Gene Expression Omnibus) repository, accession number GSE220934.

## Author contributions

HZ and XL designed and performed the experiments, also analyzed data and contributed to the writing of the manuscript. XZ, RX and YY contributed to part of data analysis. MA contributed to the consumer preference analysis and revised the manuscript. CY supervised part of the research and reviewed the manuscript. DT reviewed and revised the manuscript. XL supervised the research. All authors contributed to the article and approved the submitted version.
