# Supplementary material for: Postharvest chilling diminishes melon flavor via effects on volatile acetate ester biosynthesis
Source: Front Plant Sci. 2023 Jan 6;13:1067680. doi: 10.3389/fpls.2022.1067680 (PMC9853462; doi:10.3389/fpls.2022.1067680)
Supplement: Supplementary file 1 [file DataSheet_1.docx]

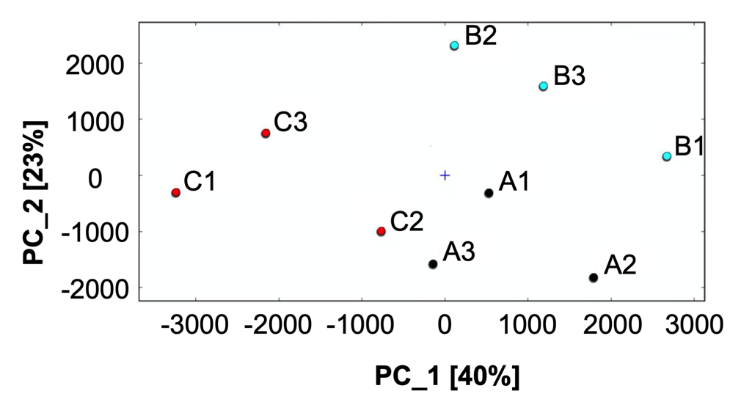


Supplementary Figure S1. Principal component analysis of differential content of VOCs Group A, B and C.


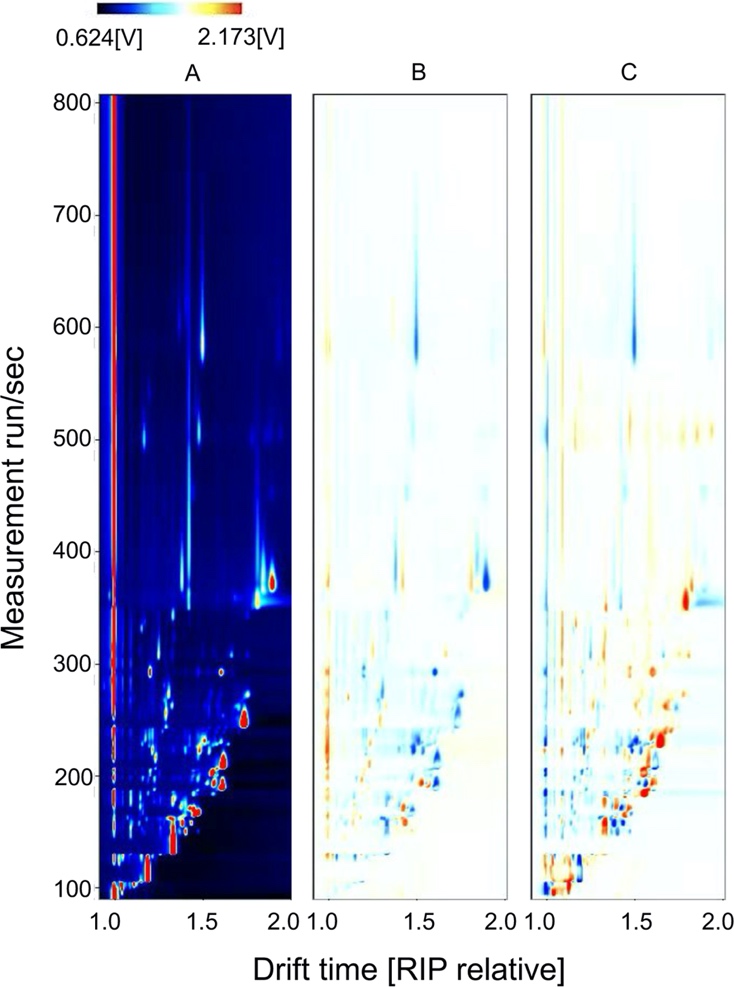


Supplementary Figure S2. Differential HS-GC-IMS images (2D topographic) of VOCs in the samples of three groups. The differential 2D ion mobility spectrums of volatiles in melon fruit after chilling followed by 1-d recovery at room temperature (Group A), after chilling only (Group B) and harvested on day 8 (Group C). A red dot signifies that the VOC showed a higher abundance in the sample of Group B and C than in Group A, whereas a blue dot signifies that the level of the VOC in the sample of Group B and C was lower than in Group A.


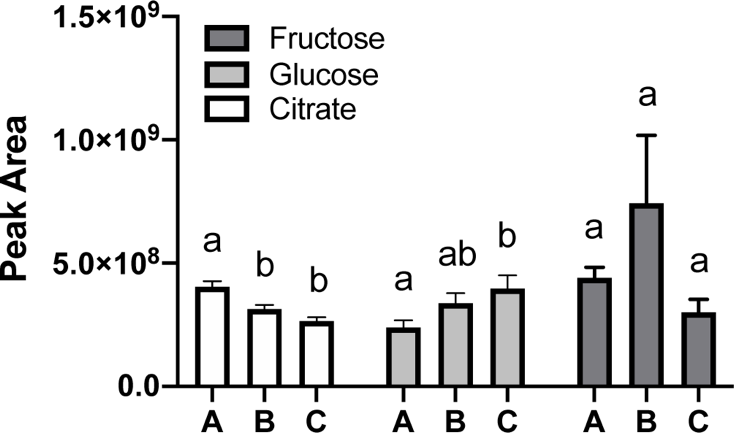


Supplementary Figure S3. Statistical analysis of fructose, glucose and citrate content in the three groups. Significant differences in sugars and acids are denoted by different letters (p-value < 0.05).


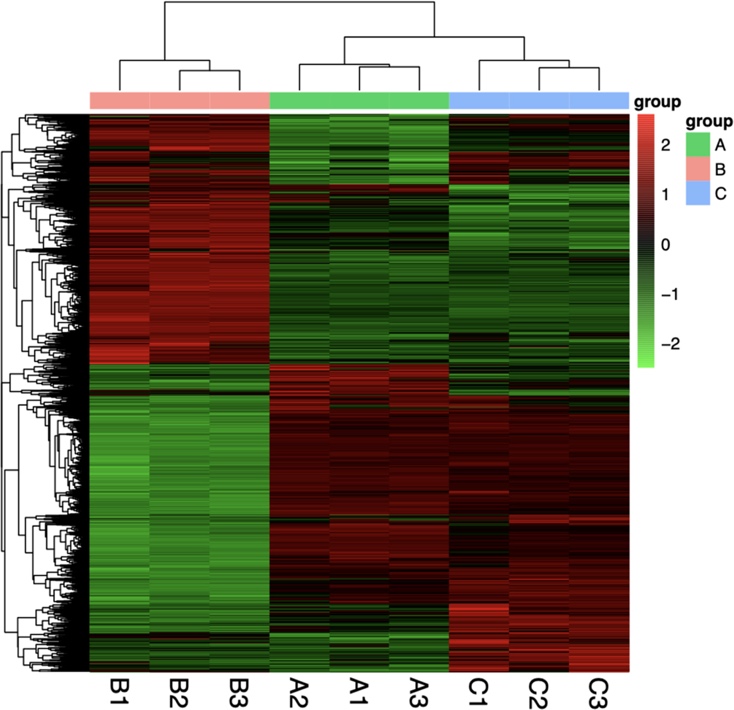


Supplementary Figure S4. A heatmap of centered and scaled FPKM of DEGs in Group A, B and C.


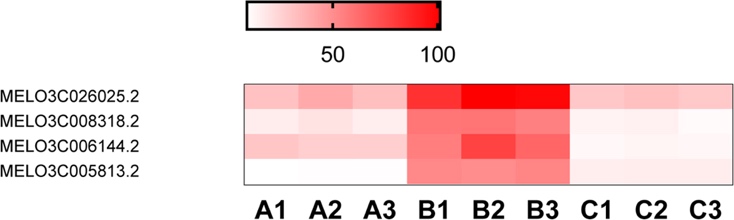


Supplementary Figure S5. A heatmap of melon dehydration responsive-element binding TFs in Group A, B and C.


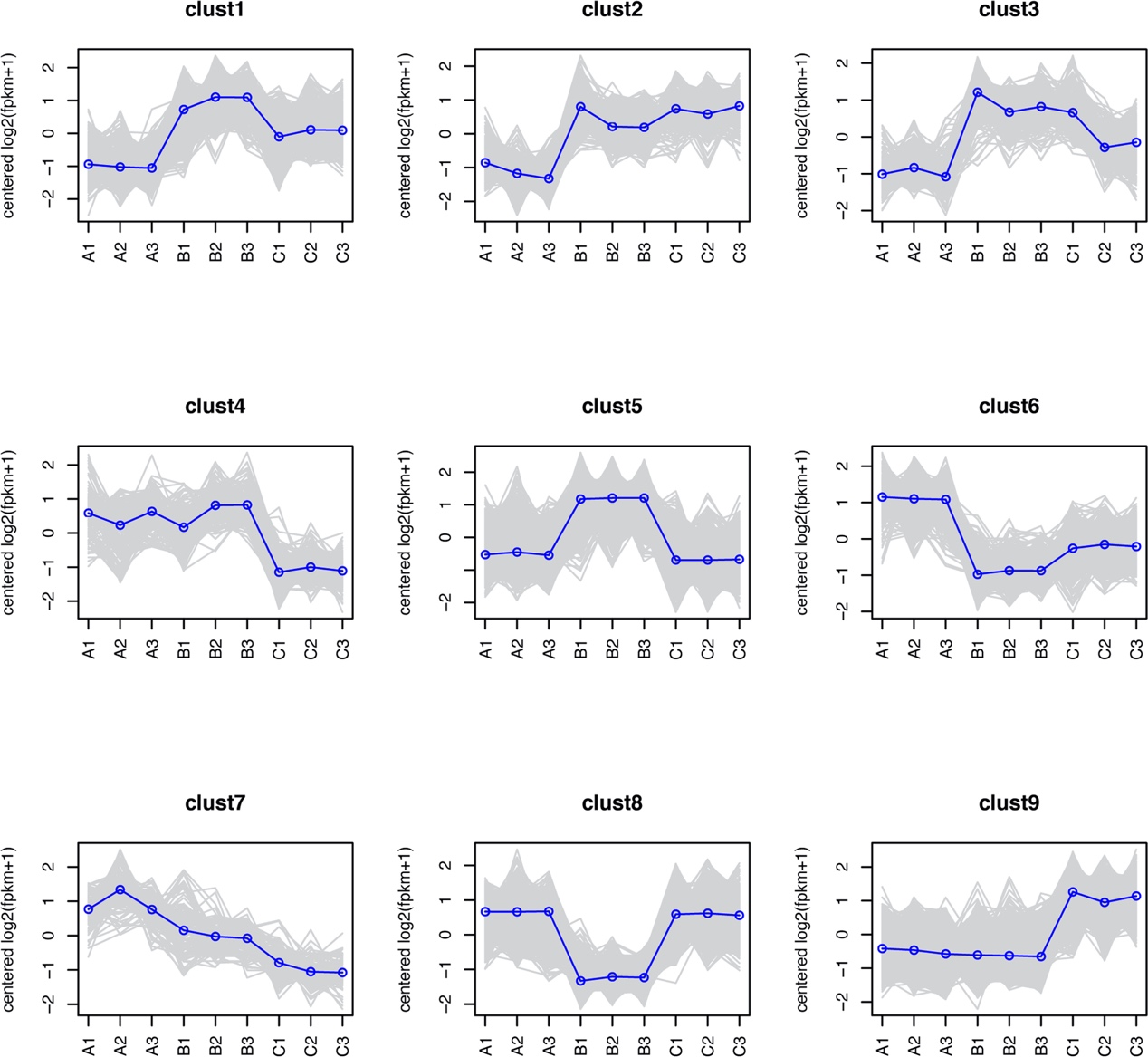


Supplementary Figure S6. DEGs identified in this study was classified into nine clusters. Grey lines indicate gene expression patterns, and blue lines indicate the average of gene expression in each cluster.


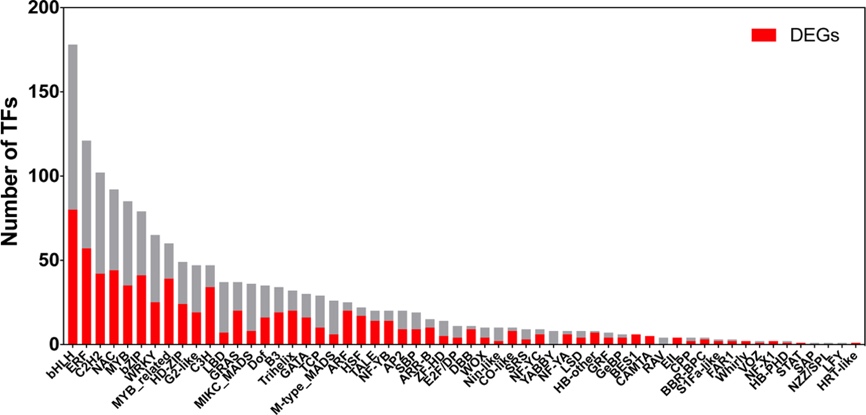


Supplementary Figure S7. Number of TF family members and differentially expressed TFs.


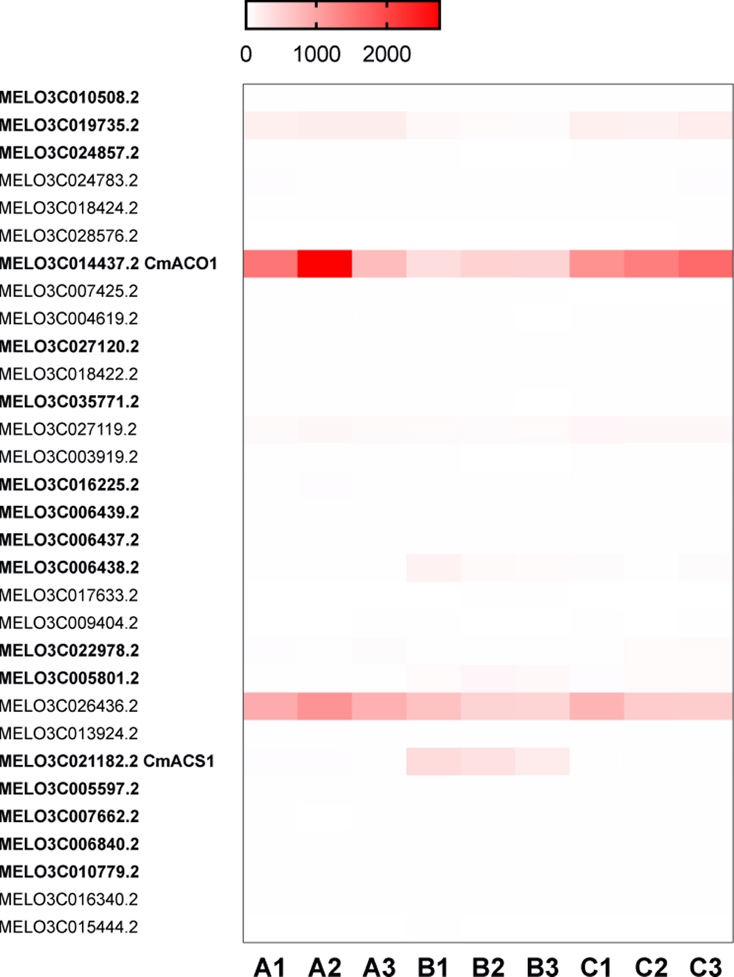


Supplementary Figure S8. A heatmap of melon ACO and ACS genes.
